# Supplementary material for: Waveguide-integrated colour centres in silicon carbide with broadband photonic crystal reflectors for efficient readout
Source: Npj Nanophoton. 2026 Jun 15;3(1):35. doi: 10.1038/s44310-026-00118-4 (PMC13269121; doi:10.1038/s44310-026-00118-4)
Supplement: Supplementary file 1 — Supplementary Information [file 44310_2026_118_MOESM1_ESM.pdf]

Supplementary Information for "Waveguide-integrated colour centres in silicon carbide with broadband photonic crystal reflectors for efficient readout"

Marcel Krumrein,[1] Julian M. Bopp,[2,3] Timo Steidl,[1] Wolfgang Knolle,[4] Jawad Ul-Hassan,[5] Vadim Vorobyov,[1] Tim Schröder,[2,3] and Jörg Wrachtrup[1,6]

[1] *3rd Institute of Physics, IQST, and Research Center SCoPE, University of Stuttgart, 70569 Stuttgart, Germany*
[2] *Humboldt-Universität zu Berlin, Department of Physics, 12489 Berlin, Germany*
[3] *Ferdinand-Braun-Institut gGmbH, Leibniz-Institut für Höchstfrequenztechnik, 12489 Berlin, Germany*
[4] *Leibniz-Institute of Surface Engineering (IOM), 04318 Leipzig, Germany*
[5] *Department of Physics, Chemistry and Biology, Linköping University, SE-581 83 Linköping, Sweden*
[6] *Max Planck Institute for Solid State Research, 70569 Stuttgart, Germany*

1. ROOM-TEMPERATURE CHARACTERISATION OF THE WAVEGUIDE-INTEGRATED V2 COLOUR CENTRES

To characterise the integrated colour centres, the emitters are excited confocally from the top with a pulsed laser diode that emits at a wavelength of 780 nm. The emitted photons are collected by a TWTF interface and detected by a superconducting nanowire single-photon detector (SNSPD). The entire setup is described in detail in previous works [1].

V2 candidates are identified in confocal scans and verified by optically detected magnetic resonance (ODMR) spectroscopy. We found ODMR peaks between 67 and 74 MHz, which is a deviation of maximal 4 MHz from the ground state zero-field splitting of 70 MHz [2]. From these values, we can conclude that the strain components within the fabricated nanobeams are not higher than $5 \cdot 10^{-4}$ [1]. A representative ODMR spectrum of one V2 colour centre is shown in the main text in Figure 3a with a peak position of (67.15 ± 0.19) MHz, a linewidth of (18.23 ± 0.36) MHz, and an ODMR contrast above 3%.

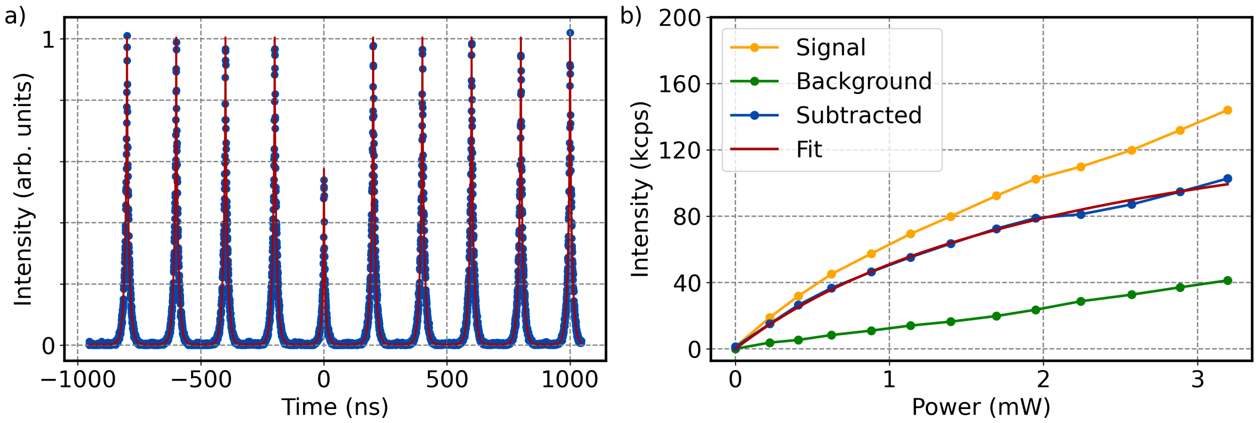

FIG. S1. Room temperature characterisation of a V2 colour centre integrated into a waveguide attached to a Dinosaur reflector at one end. The emitted photons are collected via a TWTF interface. (a) Second-order autocorrelation measurement under pulsed excitation. The peaks were fitted with a bi-exponential decay, and the area below the peaks integrated, which results in an antibunching dip of $g^2(0) = 0.4100 \pm 0.0077$. (b) Power-dependent saturation curve with a fitted saturation intensity of $I_s = (174.5 \pm 6.9)$ kcps.

The single-photon character of the V2 center is investigated by second-order auto-correlation measurements. The correlation data from a V2 center are displayed in Figure S1a revealing that the peaks follow a bi-exponential decay. For mono-exponential decay curves, the ratio of the peak amplitudes is equivalent to the ratio of the area below the peaks. This is not valid for bi-exponentially decaying signals. To obtain $g^2(0)$, we must consider the area below the peaks. For this, a multi-peak fitting routing was used to obtain the red fitting curve in Figure S1a. All peaks are fitted simultaneously with a bi-exponential function with the same decay constants for all peaks. The resulting fit matches the experimental data accurately. After dark count subtraction, the area below the peaks was numerically

integrated. The error for $g^2(0)$ is obtained by maximum error estimation: the fit parameters were varied within the 1σ error interval and inserted into the fit function. We obtain $g^2(0) = 0.4100 \pm 0.0077$ for the antibunching feature. Hence, the emitter can be considered as a photon source with a single-photon character.

Power-dependent saturation studies were performed. After subtracting the linear background, we obtain a saturation intensity of $I_{\mathrm{s,ref}} = (174.5 \pm 6.9)\,\mathrm{kcps}$. In previous works [1], we showed that waveguide-integrated V2 centers with the same background level ($g^2(0) = 0.466 \pm 0.003$) have a saturation intensity of $I_{\mathrm{s,wg}} = (224.7 \pm 8.6)\,\mathrm{kcps}$ when collecting the emission at both ends of the waveguide. With this, we can now calculate the reflectance R_{V2} of the Dinosaur reflector based on the saturation intensity by

$$I_{\mathrm{s,ref}} = \frac{1}{2} \cdot I_{\mathrm{s,wg}} \cdot (1 + R_{\mathrm{V2}}) \tag{1}$$

and obtain $R_{\mathrm{V2}} = (55.3 \pm 8.5)\%$. This is slightly lower than the average reflectance $R_{\mathrm{V2,avg}} = 65.8\%$ calculated in the reflection measurements. We attribute this deviation to a non-ideal positioning of the colour centre in the nanobeam's cross section. In conclusion, the Dinosaur reflector allows us to collect 78% of the photons compared to a collection from both ends of the waveguide.

2. ESTIMATION OF OPTICAL SINGLE SHOT READOUT

For the theoretical calculations of the optical single-shot readout in the main text, the parameters used are shown in Table I. Count rates for the bright and dark states are taken from the saturation measurement. To obtain the rates and weights, the data from [3] are taken and fitted with a bi-exponential decay. The power-dependent values were mapped to our values.

TABLE I. Parameters used for calculating the optical SSR. The count rates are taken from the saturation measurements. The weights (a' and a") and ISC rates (γ' and γ") are obtained from [3].

parameter	λ_b (kcps)	λ_d (cps)	a'	a"	γ' (1/μs)	γ" (1/μs)	T (μs)
value	105	490	0.768	0.232	1/0.48	1/3.15	10

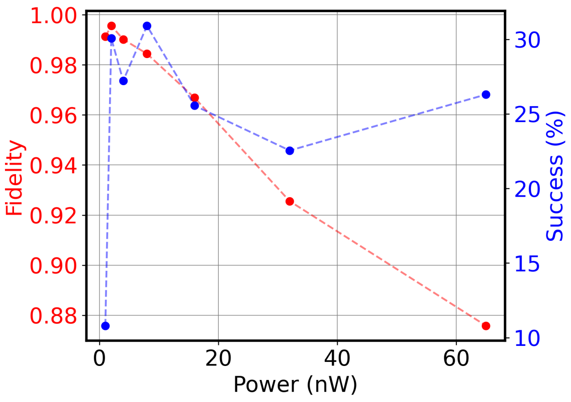

FIG. S2. Simulated fidelities and success rates for different excitation powers. The calculation procedure is explained in the main text.

The calculation of the SSR fidelity was performed for all power values. A summary of the fidelities and success rates obtained is given in Figure S2. Increasing the resonant power lowers the fidelity due to more laser-related dark counts but also does not increase the success rate, as the rate of switching into the dark state also increases [3].

3. WAVEGUIDE PROPAGATION EFFICIENCY

To assess the photon losses originating from the non-ideal propagation along the waveguide, we can consider the reference measurement $\frac{I^{\mathrm{T}}_{\mathrm{ref}}}{I^{T}_{\mathrm{sig}}}$ mentioned in the main text. Here, the transmission efficiency of a $60\,\mu$m long waveguide with

two waveguide tapers at both ends and, in total, three vertical support structures was measured. The corresponding data are shown in figure S3a. Over the wavelength range of the V2 emission (917 to 1030 nm), the transmission efficiency is between 62 and 76 %, and about 70 % on average. From [1], we know that the TWTF interface efficiency is around 90 %. As we have to consider the in- and out-coupling, the overall efficiency of the waveguide transmission is 86 %. This includes the pure propagation efficiency and the losses introduced by the support structures. As the waveguide width is about 500 nm and, thus, close to the optimal waveguide width for single-mode propagation, we expect no substantial losses from the waveguide propagation (compare with [1]). In contrast, the losses caused by the support structures cannot be neglected. Due to the geometry of the Dinosaur reflector, vertical support structures with a width of 100 nm are used here (see figure S3b). With the overall transmission efficiency of the waveguide of 86 % and the fact that the waveguide consists of three support structures, the losses per support structure can be calculated at about 5 %. This is not negligible and demonstrates the need to further optimise the waveguide geometry, in specific the support structure design, to further increase the photon count rate of the waveguide-embedded V2 colour centres. One possibility is to increase the width of the waveguide near the support structure so that the guided mode is spatially shifted farther from the waveguide edge.

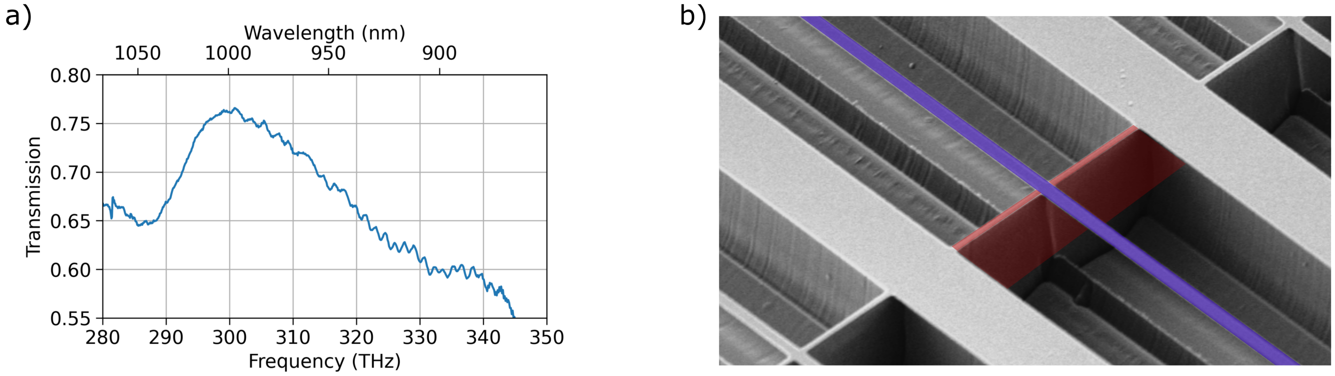

FIG. S3. Photon losses originating from propagation along the waveguide. (a) Transmission efficiency of light propagating along a 500 nm wide and 60 μm long waveguide containing of three vertical support structures. For in- and out-coupling, the TWTF interface was used. (b) False-colour SEM image of the vertical support structure. The blue colour marks the waveguide and the red colour highlights the support structure.

[1] M. Krumrein, R. Nold, F. Davidson-Marquis, A. Bouamra, L. Niechziol, T. Steidl, R. Peng, J. Körber, R. Stöhr, N. Gross, J. H. Smet, J. Ul-Hassan, P. Udvarhelyi, A. Gali, F. Kaiser, and J. Wrachtrup, Precise characterization of a waveguide fiber interface in silicon carbide, ACS photonics **11**, 2160 (2024).
[2] Ö. O. Soykal and T. L. Reinecke, Quantum metrology with a single spin-3/2 defect in silicon carbide, Physical Review B **95**, 10.1103/PhysRevB.95.081405 (2017).
[3] Di Liu, F. Kaiser, V. Bushmakin, E. Hesselmeier, T. Steidl, T. Ohshima, N. T. Son, J. Ul-Hassan, Ö. O. Soykal, and J. Wrachtrup, The silicon vacancy centers in SiC: determination of intrinsic spin dynamics for integrated quantum photonics, npj Quantum Information **10**, 10.1038/s41534-024-00861-6 (2024).
